# Polyploidy as a Fundamental Phenomenon in Evolution, Development, Adaptation and Diseases

**DOI:** 10.3390/ijms23073542

**Published:** 2022-03-24

**Authors:** Olga V. Anatskaya, Alexander E. Vinogradov

**Affiliations:** Institute of Cytology RAS, 194064 Saint Petersburg, Russia

**Keywords:** polyploidy, evolutionary conserved features, complexity, epigenetic changes, biological plasticity, adaptation, stress resistance, regeneration, carcinogenesis, aging, developmental programming, cardiovascular disease

## Abstract

DNA replication during cell proliferation is ‘vertical’ copying, which reproduces an initial amount of genetic information. Polyploidy, which results from whole-genome duplication, is a fundamental complement to vertical copying. Both organismal and cell polyploidy can emerge via premature cell cycle exit or via cell-cell fusion, the latter giving rise to polyploid hybrid organisms and epigenetic hybrids of somatic cells. Polyploidy-related increase in biological plasticity, adaptation, and stress resistance manifests in evolution, development, regeneration, aging, oncogenesis, and cardiovascular diseases. Despite the prevalence in nature and importance for medicine, agri- and aquaculture, biological processes and epigenetic mechanisms underlying these fundamental features largely remain unknown. The evolutionarily conserved features of polyploidy include activation of transcription, response to stress, DNA damage and hypoxia, and induction of programs of morphogenesis, unicellularity, and longevity, suggesting that these common features confer adaptive plasticity, viability, and stress resistance to polyploid cells and organisms. By increasing cell viability, polyploidization can provide survival under stressful conditions where diploid cells cannot survive. However, in somatic cells it occurs at the expense of specific function, thus promoting developmental programming of adult cardiovascular diseases and increasing the risk of cancer. Notably, genes arising via evolutionary polyploidization are heavily involved in cancer and other diseases. Ploidy-related changes of gene expression presumably originate from chromatin modifications and the derepression of bivalent genes. The provided evidence elucidates the role of polyploidy in evolution, development, aging, and carcinogenesis, and may contribute to the development of new strategies for promoting regeneration and preventing cardiovascular diseases and cancer.

## 1. Introduction

Polyploidy or whole-genome duplication (WGD) is widespread in nature, agriculture and aquaculture, normal physiology, regeneration, aging, and pathology [1,2,3]. WGD results from the premature termination of the cell cycle or cell fusion [2,4]. If WGD occurs in germ cells, the progeny organisms become completely polyploid, if in somatic cells, the somatic polyploidy arises in certain tissues of a given organism. Polyploidization leads to long-term consequences both in evolution (organismal) and ontogenesis (somatic) [1].

In evolution, WGD is one of the main sources for the growth of organismal complexity and evolutionary plasticity [5,6]. In ontogenesis, genome duplication can result from a trade-off between cell proliferation, stress, and tissue-specific functional load [7,8,9,10,11]. We suppose that these trade-offs are a connecting link between the role of genome duplications in normal and pathological conditions. They present a special case of the main contradiction of multicellularity (MCM) existing between the cellular and organismal levels [12]. The cellular level is presented by cell proliferation, whereas the organismal level is embodied in the control of cell proliferation and the tissue-specific functional load. The similarity between the organismal and somatic polyploidy is the increase in stress resistance, plasticity, induction of pathways of morphogenesis and longevity, epigenetic and metabolic changes.

## 2. Evolution

### 2.1. General Picture

One of nature’s greatest mysteries is how simple bacteria could give rise to such complex creatures as humans. The fundamental law of evolution (natural selection of undirected genetic changes) follows from the ‘vertical’ copying of genetic information (with small random changes). The vertical copying increases the organism’s numbers, thus resulting in competition for resources and natural selection. However, the growth of complexity does not follow from the vertical copying of information. It depends on the second fundamental copying type, which is orthogonal to the first. 

The increase in the information amount can occur due to duplication not only of the whole genome, but also of single genes, however, this review considers only the genome-wide duplication. Susumu Ohno was the first to attract attention to the role of WGD in evolution [13]. In honor of Ohno, the genes arising from the organismal polyploidization are called ‘ohnologs’ [14]. His main idea was that as a result of duplication, an additional gene copy is freed from purifying selection thereby acquiring evolutionary plasticity. He paid special attention to WGD as it completely preserves gene regulatory regions and an initial gene dosage balance, thereby providing a wide foundation for systemic evolutionary experiments. In addition, polyploidization plays a special role in the increase in complexity due to the certain properties of cellular network growth in the case of WGD as compared with the single-gene duplication [12], which will be discussed below.

Now it is firmly established that there were two rounds of ancient WGD in the vascular plant lineage (390 and 300 Mya) and in the vertebrate lineage between chordates and vertebrates (both about 500 Mya, near Cambrian explosion) [14]. In teleost fishes, there was a third WGD (300 Mya), while in salmonid fishes, a fourth (95 Mya) [15,16]. Furthermore, the more recently formed polyploid strains, species, and above-species lineages are widespread in nature, agriculture, and aquaculture. They are frequent in plants, prokaryotes, protists, fungi, invertebrates, and ectotherm vertebrates [5,17,18]. In vertebrates, the record should be placed in the Xenopus genus where 26 of 27 species are polyploids, and in sturgeons where dodecaploid species with 380 chromosomes were found [19,20]. In reptiles, there are only triploid species (and they are very rare), which propagate by parthenogenesis [21]. In birds and mammals, polyploids can also arise after conception, yet die in early development [22]. One rodent species is sometimes mentioned as polyploid, however, this attribution is probably incorrect [23]. Notably, certain model organisms are lineage-specific polyploids (yeast, zebrafish, xenopus, Arabidopsis).

### 2.2. Cellular and Molecular Aspects

One of the main problems, which new polyploids encounter, is the disturbance of chromosome pairing in meiosis. This problem is alleviated in the case of allopolyploids where polyploidization occurs due to the hybridization of two related species. In allopolyploids, the chromosomes of parental species differ and therefore conjugate separately. Probably as a result of this feature, allopolyploids are more frequently established, albeit more rarely, compared to autopolyploids [18,24]. Polyploidization is followed by rediploidization, i.e., the process of converting a polyploid chromosome set into a diploid one. Rediploidization does not mean the reversal to the original diploid state but only the re-establishment of regular chromosome pairing due to chromosome rearrangement and DNA divergence [16,20]. This process is accompanied by gene loss (due to deletion or pseudogenization), subfunctionalization, and neofunctionalization [25,26]. Subfunctionalization is a partitioning of an initial gene function (e.g., making it tissue-/development stage-/condition-specific), whereas neofunctionalization is an acquisition of a novel function. Subfunctionalization and neofunctionalization lead to gene diversification, which should be accompanied by an integration of the work of a growing number of diversifying genes, i.e., an increase in regulatory complexity [27].

In genome-wide studies, subfunctionalization can be detected by the changes in gene expression and the properties of the interactome. For example, in the yeast interactome, the proteins encoded by ohnologs have a lower number of protein interactions compared to the single-gene duplicates and genes without duplicates (i.e., genes that lost the second ohnolog) [28]. The loss of interactions was proportional to their initial number and independent of the ohnolog position in the protein interaction network. The functional analysis of the overlapping and non-overlapping interactants in each pair of ohnologs revealed a sharp asymmetry in the regulatory functions. The regulatory functions occur more frequently in the non-overlapping interactants. These facts suggest that subfunctionalization was a prevailing trend in the evolution of retained ohnologs, and was accompanied by regulatory specialization. 

The increase in regulatory complexity and elaboration of signal transduction systems is a hallmark of post-WGD evolution [29]. The predominant retention of regulatory and developmental genes subsequent to WGD can take place as relaxed selection allowed duplicated networks to be rewired, and to evolve novel functionality, thereby increasing biological complexity [5]. In the human interactome, the more ancient genes have a higher local and global centrality compared with newer genes, which indicates a gradual core-to-periphery evolutionary growth of the interactome [12]. The local centrality is the number of direct interactions of a protein (degree), while the global centrality is estimated by the number of protein-interaction paths going through a protein in the whole network (betweenness) or by the average path length to other proteins (closeness). However, there is a remarkable exception: the novel genes did not decline in the network centrality during the expansion of the multicellular organization from Bilateria to Euteleostomi (bony vertebrates) [12]. This plateau of interactome centrality is related to the series of whole-genome duplication. It indicates that in contrast to single-gene duplicates, which provide a gradual core-to-periphery network growth, in the case of WGD, the network core (that is richest in interactions) is also duplicated. Thus, the interactome complexity becomes higher in the case of growth by means of WGD, as compared with the growth by single-gene duplicates. The great diversity of vertebrates, which appeared after the two WGD rounds followed by sub- and neofunctionalization of ohnologs, can be considered as an extension of Cambrian explosion in the vertebrate lineage (reflected in the plateau of interactome centrality).

**Figure 1:**
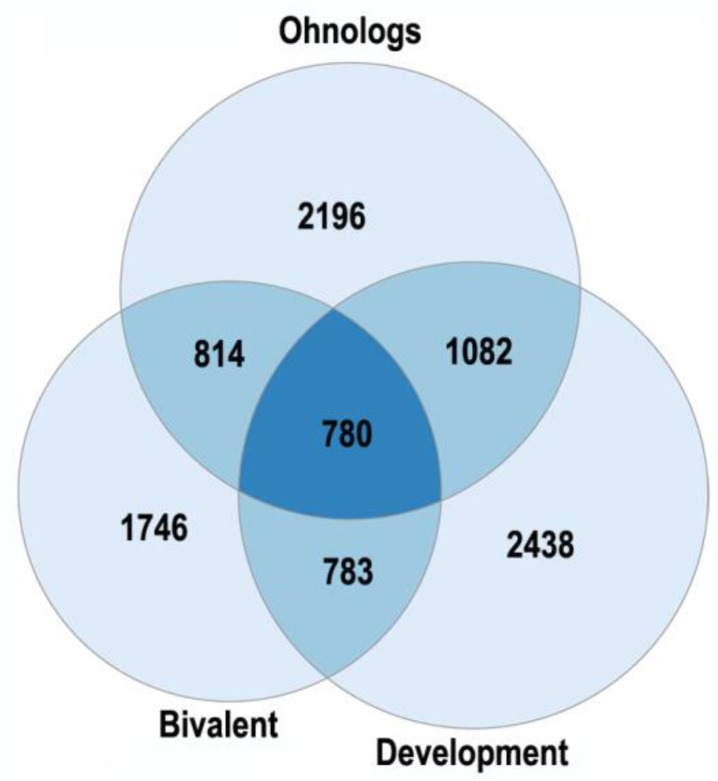
The enrichment of human ohnologs in bivalent genes and genes involved in multicellular organism development GO:0007275. Bivalent: *p* < 10^−151^, development: *p* < 10^−67^. The ohnologs (strict) were from [14]. The bivalent genes (Fantom-confirmed) were from [30]. The enrichment analysis was conducted as in [32].

**Figure 2:**
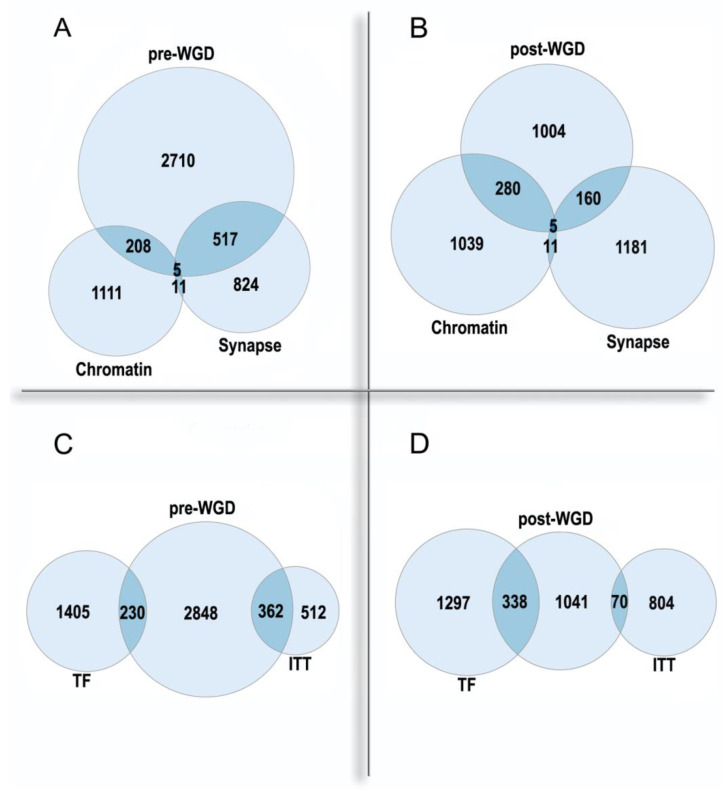
The enrichment of pre-WGD and post-WGD human ohnologs in functional gene groups. (**A**,**B**)—nuclear chromatin GO:0000790 and synapse GO:0045202. Chromatin: underrepresentation *p* < 0.01 in pre-WGD, enrichment *p* < 10^−61^ in post-WGD. Synapse: enrichment *p* < 10^−71^ in pre-WGD; enrichment *p* < 10^−8^ in post-WGD. (**C**,**D**)—transcription factors (TF) and ion transmembrane transporter activity GO:0015075 (ITT). TF: underrepresentation *p* < 10^−7^ in pre-WGD, enrichment *p* < 10^−74^ in post-WGD. ITT: enrichment *p* < 10^−56^ in pre-WGD, not significant *p* > 0.4 in post-WGD. Albeit both pre-WGD and post-WGD ohnologs are enriched in the synapse genes, there are significant differences in binomial proportions between pre-WGD and post-WGD ohnologs (*p* < 10^−15^). The pre-WGD and post-WGD ohnologs were from [33]. The transcription factors were from [34].

The human ohnologs are most strongly enriched in the bivalent genes and the genes related to development, neuronal membrane, and chromatin (Figure 1 and Figure 2). The bivalent genes, which have both activating and repressive epigenetic marks in their promoters and can switch on/off quickly, are key regulators of cellular networks [30,31]. These facts emphasize the important role of ohnologs in regulation.

The ohnologs present a unique model for the study of evolutionary speed in different functional gene groups (as WGD presents the common starting point). Albeit all ohnologs are of pre-WGD origin by definition, it is possible to distinguish those of them, which experienced an accelerated sub/neofunctionalization after the WGD, using the method of shallow phylostratigraphy (gene dating) [33]. These genes rapidly accumulated changes and can be mapped only to the post-WGD phylostrata by shallow phylostratigraphy. In other words, as a result of the rapid evolution, they lost strict orthology with the genes from phylogenetic lines branching before the WGD and retained it only with the genes from the post-WGD branches (they were called ‘post-WGD’ ohnologs). 

Importantly, the synapse and the chromatin are the most enriched Cellular Component Gene Ontology categories in the pre-WGD and post-WGD ohnologs, respectively. Thus, ohnologs are involved in both regulatory levels of the organism: the nucleome and the connectome. The pre-WGD ohnologs show enrichment in the synapse genes and underrepresentation in the chromatin genes, whereas the post-WGD ohnologs show a stronger enrichment in the chromatin genes than in the synapse genes, with significant difference in these enrichments (Figure 2A,B). The most enriched GO Molecular Function in the pre-WGD ohnologs is the ion transmembrane transporter activity, whereas in the post-WGD ohnologs it is the transcription factors (Figure 2C,D). Thus, nuclear regulome shows a faster evolution than the molecular basis of neuronal signal transmission. Possibly, this is due to the main regulatory task in the nervous system being shifted from the molecular to the cellular level, and this shift can restrict the evolution of involved genes.

In addition to the increase in complexity, polyploids show enhanced evolutionary plasticity [35]. Large-scale species radiation in a given taxonomic group typically follows polyploidization events, indicating the potential of polyploidization for speciation [36]. However, whatever evolutionary perspectives of polyploids may be, natural selection is not teleological and works only for immediate adaptation. Polyploids show a higher resistance to environmental stress and diseases [37,38]. They are especially advantageous under dramatically challenging environments [39]. Thus, many ancient polyploidization events were concentrated near crucial historical periods, such as the Cretaceous–Paleogene boundary, when an asteroid hit the Earth resulting in the drastic alteration of climate and mass extinction [36].

### 2.3. Polyploidy in Agriculture and Aquaculture Biotechnology

Polyploidy was a key factor in the domestication of crops [26,40,41]. Domesticated plants have gone through more polyploidization events than their wild relatives [40]. Genetic plasticity of the polyploid genome and multicopy genes present a special advantage for domestication [36]. Among lineage-specific polyploids are such major crops as wheat, oat, maize, cotton, potato, legumes, banana, sugarcane, oilseed rape, strawberry, coffee, mustard, tobacco. The domesticated organisms are mostly allopolyploids (i.e., the results of polyploidization associated with hybridization). Hybridization is frequently accompanied by enhanced heterozygosity and hybrid vigor, while polyploidization restores the fertility of newly formed hybrids and contributes to the stabilization of the hybrid genome, fixing both heterozygosity and new hybrid characters [41].

Allopolyploidization presents a way for new organisms’ formation. Synthetic polyploids have been employed to increase beneficial traits, such as higher fitness, disease resistance, faster growth, and larger production compared to their natural diploids [42,43]. Thus, the synthetic hexaploid wheat presents a novel source of genetic diversity for multiple biotic stress resistance [38]. Many artificial polyploids are utilized commercially in aquaculture and most of them were created from natural polyploid fishes and shellfishes, especially cyprinids and salmonids [44]. There is a competition between parental genomes in polyploid hybrids, called ‘subgenome dominance’, when the less expressed subgenome tends to delete more genes and cis-acting elements compared to the counterpart subgenome [26,41]. In extreme cases, the parental genomes can even segregate separately in germ cell lineage and a hybrid state arises de novo in each generation [45,46]. The recursive production of de novo polyploid hybrids is used when allopolyploids are sterile or lose their hybrid vigor through inbreeding depression. For instance, an allohexaploid carp is massively propagated by crossing parental species in each generation [44]. 

## 3. Evolutionary Medicine

Evolutionary medicine is a rapidly developing field [47]. There are two polyploidy-related aspects of it. Firstly, mutations and changes in expression patterns of many ohnologs are associated with various diseases and developmental disorders [14]. Secondly (and probably more important), cancer is now interpreted as an evolutionary phenomenon, and polyploidy often arises in cancer [48,49,50].

### 3.1. Ohnologs and Diseases

**Figure 3:**
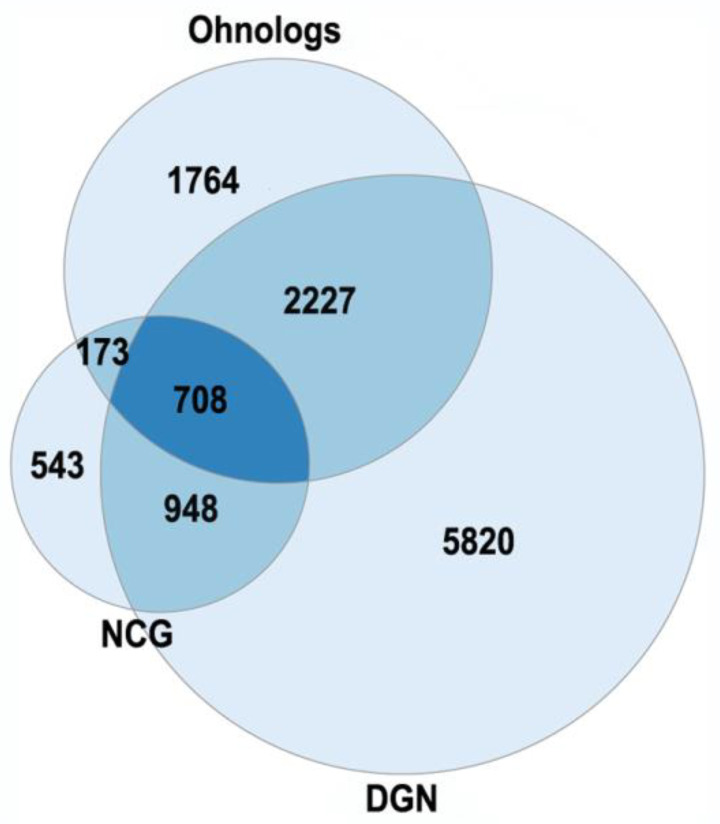
The enrichment of human ohnologs in Network of Cancer Genes (NCG) and Disease Gene Network (DGN). NCG: *p* < 10^−46^, DGN: *p* < 10^−107^. The NCG were from [54], the DGN (curated part) were from [55].

The currently existing ohnologs are in gene balance as dosage-sensitive genes [51,52,53]. Gene-dosage sensitivity has been of increasing interest as it might provide a clue for many pathologies [53]. The gene balance model states that a stoichiometric equilibrium is maintained among all of the complex gene products in a pathway, so a change in expression, mutation or copy number variation in a single gene of a pathway would be deleterious. The changes in ohnolog expression patterns are found in different types of cancers (breast, prostate, colon, thyroid, ovary, bladder, cervix, lung, uterus) and involved in tumor angiogenesis [52]. Human monogenic disease genes in the OMIM database are enriched in ohnologs [53]. Ohnologs are also highly enriched in the genes from the Disease Gene Network and the Network of Cancer Genes, which involve diseases with more complex etiologies (Figure 3).

### 3.2. Polyploidy in Cancer 

The main contradiction of multicellularity stems from its evolutionary origin, as it is between the cellular and organismal levels [12]. Cell pluripotency and proliferative potential are vital for the healthy development and longevity of multicellulars if held in check. In this case, activity at the unicellular level promotes an organism’s vitality. In contrast, runaway unicellularity results in cancer when cells tend to behave as independent evolutionary units. The atavistic theory of oncogenesis assumes that cancer is a reversal from a multicellular to a unicellular state and is frequently associated with polyploidization [50,56,57]. The genes and transcriptome modules of unicellular origin are overexpressed in human cancers, whereas multicellular genes and modules are downregulated [49,58]. 

These results were obtained on the bulk tissues and without control for cell cycle activity. Therefore the picture remained uncertain as: (i) cell cycle activity can be higher in cancer tissues; and (ii) expression of unicellular genes positively correlates with expression of cell cycle genes even if the overlapping genes are removed [59]. Yet, the upregulation of unicellular genes and the unicellular giant cluster of interactome was recently observed in the single-cell transcriptomes of various cancer types with the control for cell cycle activity [59]. Furthermore, the upregulation of unicellular genes and interactome giant cluster was shown in the invasive myeloma cells as compared with non-invasive cells. The upregulation of the unicellular giant cluster suggests that oncogenesis is not just an alteration in a few genes but the switching to ancient unicellular programs (when cells tend to behave as independent organisms). The interactome clusters have a higher interaction density within than between them, therefore they can serve as attractors (stable states of dynamic systems) of cellular programs. The unicellular cluster is denser (it has a higher inside/outside interaction ratio compared with multicellular clusters), which indicates a stronger attractor effect and helps explain why cells of multicellular organisms are prone to oncogenesis [12]. These observations suggest practical applications as certain unicellular-specific drugs can be applied for the treatment of cancer [60].

The upregulation of G2/M checkpoint genes with downregulation of G1/S checkpoint was found in polyploid breast cancer cells (called ‘G2/M vs. G1/S checkpoint inversion’) using single-cell transcriptomes with the control for cell cycle activity [59]. Probably, the hyper-activation of the G2/M checkpoint (which can be caused by DNA damage) retards the G2/M transition. In accordance with this suggestion, the genes belonging to G2/M transition were downregulated in polyploid cells, whereas the genes belonging to G1/S transition were upregulated (‘inversion of G2/M vs. G1/S transition’). The cytokinesis-related genes were downregulated [59]. 

Polyploidy results from the overall instability of metabolically stressed cancer cells [50,61]. In single-cell transcriptomes, the cancer cells show a higher scatter around the regression line of expression of the unicellular genes and interactome giant cluster on the cell cycle genes compared with normal cells [59]. This observation demonstrates a greater variability of cancer cells caused by their deviation from a stable, ‘canalized’ line (sensu [62]) of normal cells. Polyploidy facilitates chromosomal instability, which leads to aneuploidy and chromosome abnormalities increasing the probability of transformation [50,63]. Notably, under the influence of a carcinogenesis promoter (12-O-tetradecanoylphorbol-13-acetate), human lymphocytes in primary culture continue DNA synthesis even when mitosis is blocked by colchicine or cytokinesis is blocked by cytochalasine, thereby forming cells with high ploidy [64]. This indicates the role of the loosening of cell cycle control in pathological polyploidization. Polyploidy in tumors is associated with resistance to radio/chemotherapy and poor prognosis [65,66].

Intriguingly, polyploidization by fusion of different cell types (epigenetic hybridization), which is frequently observed both in normal and cancer tissues [4,63], strikingly resembles allopolyploidization (genetic hybridization) in the evolution and agri/aquaculture described above. Cell fusion plays a crucial role in several physiological processes, including wound healing and tissue regeneration [4]. Thus, the bone marrow-derived stem cells (BMSCs) could adopt the specific properties of a different organ by cell fusion, thereby restoring organ function [4]. Similar to the allopolyploid organisms, the hybrid polyploid cells acquire stress resistance (e.g., chemo- and radiation-resistance) and adaptive plasticity. For instance, fusion with motile leucocytes such as macrophages renders cancer cells an ability to migrate, which plays a major role in metastasis [63]. Furthermore, cell fusion can be a mechanism of cancer stem cell formation [63]. Cancer stem cells present an important problem as they are resistant to therapy and serve as a source of new cells after the reduction in tumor mass by treatment [63,67]. 

**Figure 4:**
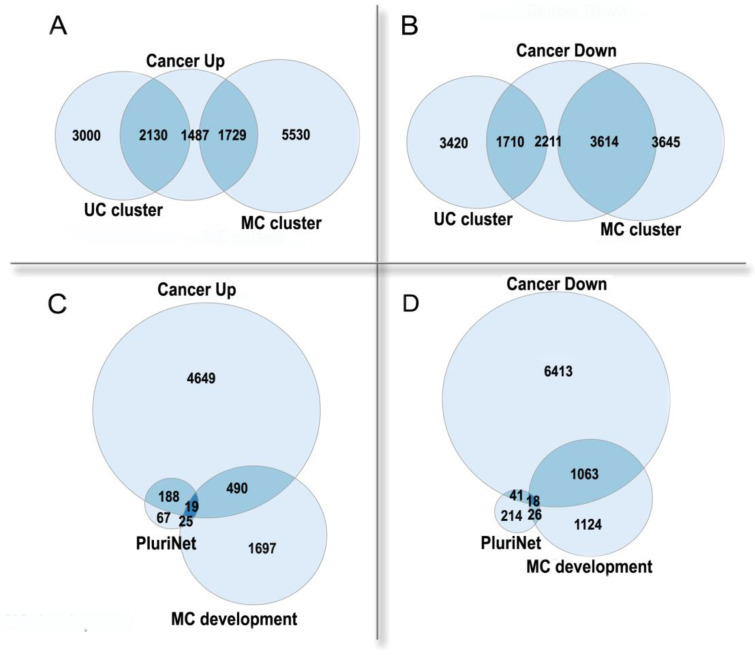
The enrichment of human genes, which are up- or down-regulated in polyploid cancer cells as compared with diploid cells of the same cancer, in functional gene groups. (**A**,**B**)—unicellular (UC) and multicellular (MC) giant clusters of interactome.UC cluster: enrichment *p* < 10^−157^ in Up, underrepresentation *p* < 10^−15^ in Down.MC cluster: underrepresentation *p* < 10^−14^ in Up, enrichment *p* < 10^−145^ in Down. (**C**,**D**)—pluripotency signature (PluriNet) and regulation of multicellular organismal development GO:2000026 (MC development). PluriNet: enrichment *p* < 10^−51^ in Up, underrepresentation *p* < 10^−11^ in Down. MC development: underrepresentation *p* < 10^−5^ in Up; enrichment *p* < 10^−28^ in Down. The up- or down-regulated genes in polyploid cancer cells were from [69], which was based on the analysis of about ten thousand cancer samples. The UC and MC cluster genes were from [12], the PluriNet genes were from MSigDB [70].

Thus, polyploidy in cancer is associated with a poor prognosis as a result of resistance to stress, stemness, and an enhanced migration ability [50,63]. Paradoxically, albeit in normal tissues polyploidization frequently accompanies differentiation [1,68], polyploidy in tumors is associated with the features of fetality and stemness [50,63]. This paradox can be solved if considering cancer from an evolutionary viewpoint and comparing it with organismal polyploidy. Polyploid cancer cells show a general increase in adaptivity, which is reminiscent of the rapid growth, stress resistance, and the evolutionary plasticity of polyploid organisms. Stress caused by diseases, which results in the formation and survival of polyploid cells, can be considered as an analog of environmental stress conferring an adaptive advantage to polyploid organisms. In the polyploid cancer cells, the expression of the unicellular giant cluster of interactome is upregulated, whereas the multicellular cluster is downregulated, even as compared with diploid cells from the same cancer (Figure 4A,B). The pluripotency signature (PluriNet) is upregulated, whereas the genes involved in the regulation of multicellular organismal development are downregulated (Figure 4C,D). These facts indicate that the polyploidization of cancer cells enhances their unicellular properties and proliferative potential, at the same time reducing the multicellular-level control.

## 4. Somatic Polyploidy

### 4.1. Somatic Polyploidy Is a Way of Adaptation to Stress 

Somatic polyploidy was found in the tissues of all multicellular organisms (including algae, mosses, lichens, vascular plants, invertebrates, and vertebrates), which points to its adaptive value [3,5]. In human and warm-blooded animals, polyploidy can be a part of normal postnatal morphogenetic programs and can be a manifestation of response to pathological stimuli and diseases. Thus, polyploid cells arise in normal organogenesis of heart, neuronal glia, cerebellum, neocortex, retina, liver, placenta, blood vessels, skin, blood, and other organs [3,22,71,72,73,74] and in atherosclerosis, neurodegenerative disorders, cardiovascular diseases, wound healing, inflammation, diabetes, cancer, and other pathologies [1,7,8,72,74,75,76,77,78,79,80]. Despite the prevalence in normal physiology and pathology, the functional significance of polyploidy still is not clear. Contrary to the old hypothesis, polyploidy is not necessarily required for differentiation and does not have a strong effect on proliferation [81]. Moreover, polyploidy is far from always associated with the increase in protein cell content in proportion to the number of genomes, which makes its role in the regulation of cell and organ size ambiguous [81,82].

Some researchers believe that somatic polyploidy is harmful as it slows down proliferation, inhibits regeneration, reduces cell functionality and promotes genetic instability [69,83,84,85,86,87,88]. Others think that additional genomes are useful as they enhance cell function due to the acceleration of metabolic processes, protein synthesis, regenerative properties, and protect cells from oncogenic transformation [1,3,89,90]. There are also opinions that genomic duplications are neutral as polyploid cells can be an approximate equivalent to the corresponding number of diploid cells [91]. 

The lack of consensus may originate from the fact that polyploid cells exhibit different properties in tissues with different growth activity and differentiation states. For example, in the growing heart and liver, polyploidization of cardiomyocytes and hepatocytes occur as a result of restriction of the last cell cycle phases (cytokinesis and karyokinesis) and is associated with a slowdown of proliferation [72,92]. In the heart and liver of adult mammals, where the mitotic activity of cardiomyocytes and hepatocytes is extremely low, de novo polyploidization (stimulated by hyperfunction or stress) occurs due to cell cycle reactivation and DNA synthesis [72,77]. In this case, the cells also lose the last phases of the cell cycle, however, the cell cycle activity in these cells is higher than in the resting diploid cells [72]. 

Despite different manifestations of polyploidy in various biological contexts, there is one important common feature that was previously attributed only to polyploidy in pathology—the association between polyploidy and stress. Extensive analysis of the recent literature indicates that polyploidy is always associated with stress, both in physiologic and pathologic contexts. Thus, in normal mammalian tissues, genome accumulation coincides with critical periods of postnatal growth when cells are forced to combine proliferation and differentiation and to undergo physiological stress [2,7,9,72,93]. Cardiomyocytes accumulate genomes during metabolic maturation coinciding with ROS overproduction and genome instability due to lamina reorganization and transition to the oxygen-rich postnatal environment [94,95]. Macrophage polyploidization during inflammation is also caused by DNA damage [96]. Hepatocytes undergo polyploidization in development in the course of transition from liquid to solid food when the physiological microenvironment is particularly genotoxic [2]. Trophoblast cells duplicate genomes along with decidualization accompanied by the increase in secretory activity and invasiveness [97,98,99]. 

Stress promotes genome accumulation in quiescent, dormant, and proliferating cells. In quiescent cardiomyocyte and hepatocyte from the adult human and mouse and in the dormant cancer cells that survived treatment, stress induces DNA re-replication resulting in polyploid cell formation [2,77,79,100,101]. In the proliferating cells, physiological stress associated with genome instability can promote polyploidy via the premature cell cycle and disrupted cell differentiation [94]. This phenomenon was described in cardiomyocytes from the hypoplastic left ventricle in the human and neonatal mice [94,102]. It was also observed in cardiomyocytes and hepatocytes of neonatal rats that survived severe inflammatory stress [7,9,75], and in drosophila epithelial cells involved in wound healing [1,103]. Thus, evidence coming from various objects and fields of research suggest that polyploidy is a way of adaptation to stress and related complications like increased ROS production and DNA instability. 

### 4.2. Polyploid Cells Reduce the Functional Capacity of the Organ

A comparison of polyploidization in hepatocytes and cardiomyocytes of mammals and birds, which differ in functional potential of the heart and liver, indicated how polyploid cells can affect organ function [71,82,104]. For the heart, the functional potential was estimated by the organ mass relative to body mass (heart index), for the liver, by the metabolic scope (i.e., the difference between the basal and maximal metabolic rate). The investigation of 36 species of birds and 30 species of mammals showed that the most severe polyploidization of hepatocytes and cardiomyocytes was observed in the animals with a small metabolic scope and a low heart index, which means that polyploidization reduces the functional potential of the organ [71,82,104].

Paradoxically, organ functional potential in adults inversely correlated with its functional load in neonates. The data obtained with 30 species of wild mammals belonging to six orders indicated that an organ, which works intensively in the adult state, is subjected to a low workload during ontogenesis and plentifully furnished with resources [70]. On the contrary, an organ with low functional potential in the adult state starts to work intensively just after birth and experiences a shortage of resources during growth. This paradox can be explained by the assumption that an organ with high functional potential should be formed under beneficial conditions. Cell ploidy in the adult state positively correlated with the neonatal functional load (as polyploidization is caused by the overlapping of cell function with proliferation during growth). The data obtained with 36 bird species that are either mature or immature at hatching confirmed the positive correlation between adult cardiomyocyte ploidy, maturity and mobility at birth, and cardiac functional load during growth [104,105].

The best examples illustrating these relationships are the couples of sedentary and athletic species with similar weights and differences in the maturity at birth and the organ functional load during neonatal development (when polyploidization begins). Thus, in an excellent athlete wolf (*Canis lupus*), that is immature-born and has low cardiac functional load during active growth, the average cardiomyocyte ploidy is 4.1 *n*, and relative heart mass is 0.8%. The corresponding values for a sedentary swine (*Sus scrofa*) that is mature-born and mobile from birth, are 8.5 *n* and 0.25% [71,82]. Accordingly, an athletic Cooper’s hawk (that is immature and immobile at hatching, yet able to fly incessantly for 10 h in the adult state) has only 4.1 *n* per cardiomyocyte and a relative heart mass of about 1.0%, whereas the hen (that is mature and mobile at hatching and can be in the air only for a few seconds in the adult state) has 6.7 *n* per cardiomyocyte and a relative heart mass about 0.4%. The obtained data contradicted the widespread opinion that polyploidy enhances organ function.

### 4.3. Functional Load Can Control Polyploidization during Postnatal Organogenesis of Heart and Liver

In slowly renewing or terminally differentiated organs of warm-blooded animals (e.g., heart and liver), neonatal genome accumulation is irreversible [77,106]. The relationship between polyploidy and the decrease in the organ functional potential makes the factors regulating genome accumulation in somatic cells particularly important. To elucidate this point, the key features of early postnatal development (growth rate, degree of maturity at birth, type of development, metabolic rate) were compared in the large-scale studies of mammals and birds with different polyploidization of cardiomyocytes [71,82,104].

It is well established that neonatal genome accumulation is irreversible as the heart and liver cells are not replaced during the life span [77,106,107]. Recent studies using labeled isotopes (15N and 14C) have confirmed that in humans and mice, cardiomyocytes and hepatocytes can be of the same age as the individual himself [77,106]. The largest number of cells with an extreme life span has been found in the heart, where a complete set of cardiomyocytes is established in postnatal growth and remains stable throughout life [77,106]. Thus, in many mammalian species, cardiomyocytes accumulate genomes during the period of milk feeding (for example, in a rat from seven to fourteen days after birth, in a pig—from a week of age to two months), in birds cardiomyocyte genome accumulation proceeds during the interval from birth to maturation [71,82,104]. In humans, polyploidization occurred mostly from birth to 11 years [77,108]. Consequently, factors regulating this process in adult animals should be sought in early postnatal development (in childhood). The studies of cardiomyocyte ploidy in more than 80 species of birds and mammals indicated that the degree of polyploidization reflects cardiac functional load during growth [82,104,105,109]. Cardiomyocytes of precocious mammals and birds, which are relatively mature and mobile from hatching or birth, contain 1.6 fold more genomes than cardiomyocytes of altricious species of similar weight, which are helpless at hatching or birth and show weak mobility during growth [82,104]. Thus, cardiac functional load during critical developmental windows is important for polyploidization.

## 5. Ploidy-Associated Transcriptome Features Are Related to Stress Response, Metabolism, Morphogenesis, and Longevity

The association between polyploidy, adaptation to stress, carcinogenesis, and decrease in organ functional potential suggests that genome accumulation is involved in the regulation of gene expression. The analysis of gene expression in the human and mouse hepatocytes, cardiomyocytes, trophoblast cells, neurons, adipose mesenchymal stem cells, interstitial cardiac stem cells, drosophila epithelial cells, and various types of cancer cells indicate that polyploidy can exert both common and specific effects [7,69,73,78,83,84,110,111,112]. The common effects are the induction of biological pathways related to stress response (i.e., abiotic, biotic, hypoxic, oxidative, genotoxic, and inflammatory), response to DNA instability, and drug resistance [7,57,69,72,83,87,113,114,115]. Polyploidy also activates the signaling cascades involved in embryogenesis (including Notch, TGFb, Hippo, Myc, EGFR, and WNT) and the growth-related gene modules implicated in stemness, DNA synthesis, glycolysis, and ribosome biogenesis [1,7,11,72,74,77,78,110,111,112,116]. The ploidy-inhibited modules are mostly involved in apoptosis, immunity, and aerobic metabolism [11,74,117].

Importantly, the main ploidy-associated features are similar to those observed in the long-living animal species and mutants. For example, an enhanced response to hypoxia, the induction of DNA repair pathways, proliferation, morphogenesis, glycolysis, adaptation to stress, ribosome biogenesis, as well as the suppression of apoptosis and aerobic metabolism, were found in the mole rats and in the long-living mutants of the mouse, nematodes, drosophila, and yeast [118,119]. Somatic polyploidy increases the lifespan of cells in the tissues and cultures, mainly due to the increased resistance to apoptosis, DNA damage, and genetic instability [74,113].

### 5.1. Ploidy-Associated Transcriptomic Features Are Evolutionary Conserved

The fundamental nature of the above-described properties of somatic polyploidy is supported by the fact that similar gene modules are enriched in the ohnologs of polyploid organisms, such as the yeast, Arabidopsis, amphibians, and bony fishes [5,120,121]. These modules include ribosome biogenesis, transcription, proliferation, glycolysis, adaptation to hypoxic and oxidative stress, and negative regulation of apoptosis [120,122]. Similarly, certain modules suppressed in the case of somatic polyploidy (aerobic respiration, signal transduction, transport, apoptosis, and immune response) in the same polyploid organisms contain the genes that have lost their duplicates (i.e., they are non-ohnologs). This fact emphasizes the common features between organismal and somatic polyploidy. Probably, the adaptation to stress, which is important in the case of somatic polyploidy, plays a role in the evolutionary fixation of organismal polyploidy and retention of ohnologs.

**Figure 5:**
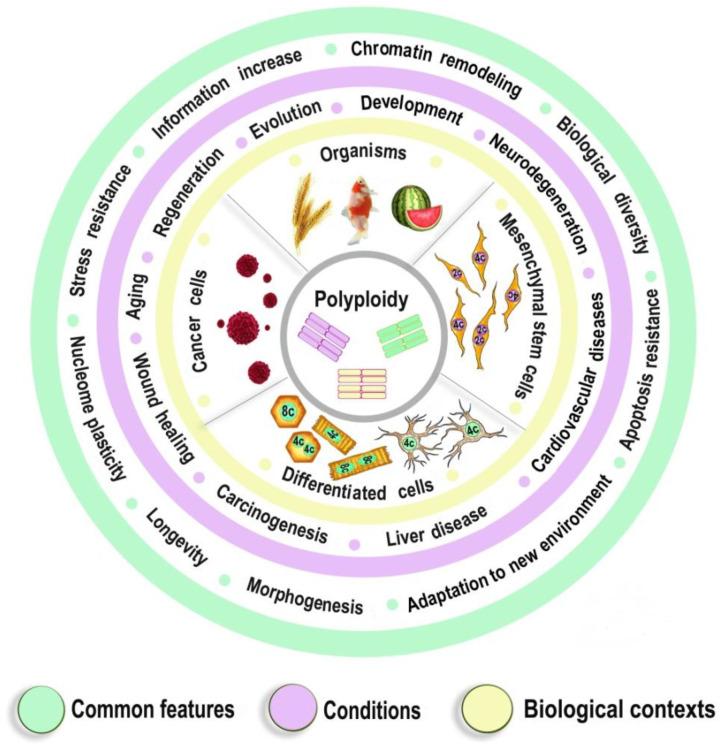
The most important Common features of polyploidy found at various physiological conditions and in different biological contexts.

Thus, the properties of polyploid genomic machinery are conservative in phylogenesis and ontogenesis and are aimed at improving survival under new conditions, plasticity, adaptability to stress, and increasing longevity. The involvement of genomic duplication in the regulation of developmental programs, life expectancy, and adaptation to stress indicates the importance of polyploidy in the physiological and pathological processes, which affect postnatal morphogenesis and adaptation (including developmental programming of widespread diseases, tissue regeneration, and carcinogenesis). Figure 5 illustrates the most important common features of polyploidy existing at various physiological conditions and in different biological contexts.

### 5.2. The Epigenetics of Ploidy-Associated Transcriptomic Features

The data from various fields of research indicate that polyploidy is associated with epigenetic changes at different levels of genome organization, which leads to chromatin remodeling and genome instability. The most obvious effect of polyploidy is the reduction in the nuclear surface-to-volume ratio resulting in a partial loss of nuclear lamina (NL) interactions with the lamina-associated domains (LADs), which increases the competition for NL contacts [123,124]. The LADs reside mostly in inactive chromatin (heterochromatin) and comprise a substantial part of the genome (about 35%) [125]. Detaching from the NL and moving inside the nucleus, LADs contribute to the opening of chromatin [123]. This is in agreement with the fact that the upregulation is stronger than the downregulation in the ploidy-associated changes in gene expression [126].

Polyploidy is also accompanied by a decrease in lamin B expression, regulating chromosome centromere attachment to mitotic spindle filaments and metaphase progression [127]. Inactivating of Lamin B decreases metaphase progression and increases genetic instability due to the impaired attachment of chromosomes to the mitotic spindle fibers [95,127]. Accordingly, the association between polyploidy and chromatin decompactization under stress was well documented for cardiomyocytes and hepatocytes [76,128]. At the lower levels of genome organization, polyploidy can alter global patterns of DNA methylation, microRNA expression, and histone modification in mammal, insect, and plant cells [1,2,98,126,129,130]. Polyploid cells show higher expression of bivalent genes, which harbor both activating (H3K4me3) and repressive (H3K27me3) chromatin domains [78,112]. This effect strikingly resembles strong enrichment of bivalent genes in the ohnologs in the case of organismal polyploidy (Figure 1A). The bivalent genes allow the cell to quickly change gene expression patterns, facilitating the formation of adaptive self-organizing regulatory networks [78]. Possibly, bivalent genes are the key regulators, which determine the common features of organismal and somatic polyploidy.

## 6. Polyploidy Meets the Hallmarks of Developmental Programming of Adult Diseases in Slowly Renewing or Terminally Differentiated Organs

Growth retardation, inflammation, malnutrition in pregnancy, infancy, and childhood are associated with the increased risk of cardiovascular diseases, hypertension, stroke, type 2 diabetes, and neurodegenerative disorders [131,132,133,134]. This phenomenon was termed as Developmental Origin of Health and Disease (DOHAD) hypothesis [135]. The link between conditions of early postnatal development and human health decades later was suggested to be the consequences of developmental plasticity, the phenomenon when one genotype can give rise to a range of different morphological or physiological states in response to different environmental conditions during development [136]. This topic belongs to the preventive medicine considered as the medicine of the future [137].

It is well established that developmental programming operates via epigenetic changes in chromatin architecture, DNA methylation, microRNA expression, histone modifications, and others [138,139,140,141,142,143]. We hypothesized that somatic polyploidy can be one of the epigenetic mechanisms of developmental programming in slowly renewing and terminally differentiated organs. This suggestion is based on the similarity between the properties of polyploidy and the mechanisms of developmental programming described in the literature [131,132,140,141,144].

**Figure 6:**
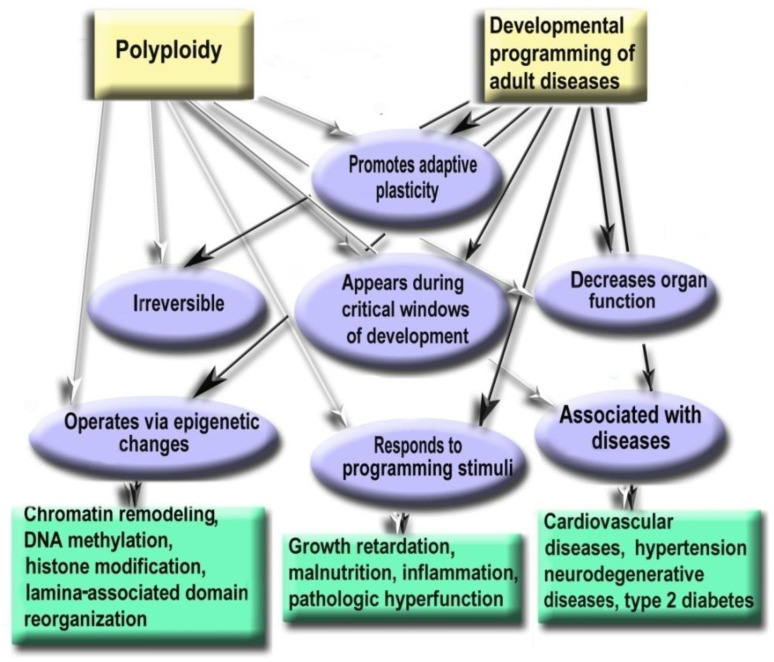
Polyploidy and developmental programming of adult diseases show similar properties.

Thus, polyploidy is similar to the mechanisms of developmental programming by the following hallmarks: (1) Promotes adaptive developmental plasticity; (2) Appears during critical developmental window; (3) Irreversible, therefore changes the organ structure and function permanently; (4) Decreases organ function and is involved in the trade-off between proliferation and function; (5) Responds to the main programming stimuli (including adverse growth conditions, growth retardation, pathologic functional load, inflammation, malnutrition, and others); (6) Regulates gene expression via the same epigenetic mechanisms as developmental programming; (7) Associated with the diseases that may arise as a result of ontogenetic programming (cardiovascular disease, hypertension, neurodegenerative disease, type 2 diabetes). Below we consider these points in more detail. Figure 6 demonstrates similar properties of polyploidy and developmental programming of adult diseases phenomenon.

1. Polyploidy helps to cope with the adverse environments via the augmentation of stress resistance and adaptation through epigenetic mechanisms [3,78,112]. Furthermore, it is one of the most variable characteristics of somatic cells. The degree of polyploidization in homologous organs shows large across-species diversity. The percentage of cardiomyocytes with polyploid nuclei varies several folds in mammals of similar weight. For example, about 50% of human cardiomyocytes contain nuclei with 4, 8, 16, or even 32 genomes, whereas cardiomyocytes of the grey wolf or reindeer show only about 1% of cells with polyploid nuclei [71,82]. Accordingly, cardiomyocyte ploidy also varies between individuals of the same species. The mean ploidy in the normal human heart varies from about 4× to 10× [77,100,108]. Thus, polyploidy is characterized by the degree of biologic plasticity similar to the renowned factors of ontogenetic programming.
2. Polyploid cells (e.g., cardiomyocytes, megakaryocytes, hepatocytes, pancreacytes, vascular epithelial cells, retina epithelium) appeared in the perinatal and early postnatal ontogenesis [9]. These periods are characterized by high biological plasticity and coincide in time with the critical periods of development [131,132].
3. Cells of slowly renewing organs, including neurons of neocortex and cerebellum, cardiomyocytes, and hepatocytes, which accumulate additional genomes in infancy, childhood, and pre-pubertant period, retain the increased genome amount throughout their lives, regardless of environmental conditions [9,72,77,100,145].
4. Polyploidization is associated with a decrease in organ functional potential [71,82,104,109]. This decrease probably originates from the involvement of polyploidy in the trade-off between proliferation and function that is also a sign of the developmental programming of adult diseases factor [71,107,131].
5. The level of ploidy, particularly in cardiomyocytes, responds to the well-established stimuli of developmental programming (including adverse growth conditions, increased functional load, inflammation, and malnutrition) similarly in the various species and various cells [9,71,132,141]. For example, in mammal hepatocytes, cardiomyocytes, retinocytes, and drosophila somatic cells, polyploidy is associated with the increased response to stress, activated pathways of morphogenesis and glycolytic metabolism, and the weakened aerobic metabolism and apoptosis [3,11,112,146].
6. Polyploidy is associated with epigenetic changes at various levels of genome organization leading to chromatin remodeling and genome instability [95,123,124]. The association between polyploidy and chromatin decompactization under stress was well documented for cardiomyocytes and hepatocytes [76,128]. Polyploidy can alter global patterns of DNA methylation, microRNA expression, and histone modification in mammalian, insect, and plant cells [1,2,78,95,112,127,129]. Polyploid cells show higher expression of bivalent genes, which harbor both activating (H3K4me3) and repressive (H3K27me3) chromatin domains, allowing rapid switching between cellular programs [112]. Overall, ploidy-associated transcriptomic changes occur through the same epigenetic mechanisms as in the developmental programming of health and disease, including chromatin remodeling, DNA methylation, histone modification, and others.
7. Excessive polyploidization can be associated with the diseases that usually originated from the developmental programming, including cardiovascular disease, hypertension, neurodegenerative disease, type 2 diabetes, metabolic syndrome, and others [9,74,77,131,132,133,134].

### Experimental Studies Confirm the Role of Polyploidy in the Developmental Programming of Health and Disease

Recent experimental and clinical studies confirm that polyploidy can be involved in the developmental programming of adult diseases. The most convincing evidence was obtained for cardiovascular diseases that are the most susceptible to developmental programming. Thus, studies in sheep indicated that pre-term birth irreversibly increases the percentage of polyploid mononuclear cardiomyocyte and induces DNA damage, fibrosis, and lymphocytic infiltration [145]. In humans, pathologic hemodynamic load during postnatal growth permanently increases cardiomyocyte ploidy and decreases cardiac performance [79,92,95,100,101,147,148]. The inflammatory stress caused by gastroenteritis in the rat resulted in cardiomyocyte hyperpolyploidization, long-term atrophy, and cell remodeling [9,75]. The experimental model of gastroenteritis was used as gastroenteritis triggers developmental programming factors, including inflammation, growth retardation, and malabsorption, and as gastroenteritis is a major cause of diseases in toddlers, infants, and children [149,150,151]. Both types of neonatal gastroenteritis cause irreversible excessive polyploidization, long-term atrophy, and remodeling of cardiomyocytes [9,10,75]. Altogether, these data indicate that polyploidy can be involved in developmental programming as it is irreversible, responds to programming stimuli during the critical period of development, changes cell phenotype, and weakens cell function, thus meeting all basic criteria of developmental programming.

## 7. Genome Duplication in Regeneration and Aging

Polyploidization is an important way to preserve cell function and survival under stressful conditions (e.g., necrosis, inflammation, toxic stress, aging, wound healing, and pathological stress) [1,113]. This happens when it is impossible to restore the tissue by the proliferation of diploid cells (e.g., during regeneration and maintenance of organs consisting of terminally differentiated cells, including heart, liver, kidney, and brain). In these slowly renewing organs, polyploidy may be the only way to maintain functionality under stressful conditions [72].

The participation of polyploidy in regeneration is a part of an evolutionarily conservative response to damage [74]. This may be due to the increased resistance and efficiency of polyploid cells compared to diploid ones, which is particularly important during restoration. When there is a need to quickly restore function, polyploidy helps to increase cell size (although often not proportionally to the number of genomes), bypassing the energy-consuming mitosis associated with the reorganization of cytoskeleton, disruption of intercellular contacts and tissue architecture [72]. It is known that polyploidy is associated with the switching of metabolism to the energy-saving mode [8,152]. This is especially evident in the heart, where polyploidy caused by stress and hyperfunction leads to the replacement of myosin heavy chain α (fast, adult, and ATP costly) with myosin heavy chain β (slow, embryonic, and ATP economical) [75,153]. This relationship was confirmed in the experimental models of heart disease, as well as in hypertensive heart disease, dilated cardiomyopathy, myocardial infarction, and ischemia [154].

The ability of polyploid cells to maintain function in conditions of energy deficiency probably allows some species to adapt to extreme hypoxia. For example, about 80% of the cardiomyocyte nuclei in the naked mole rat (*Heterocephalus glaber*) contain four or more genomes [147]. Notably, this rodent lives under toxic conditions with a low oxygen concentration and has the longevity of 32 years, which is about tenfold greater than the mouse [155]. At the same time, in other rodents of similar weight, cardiomyocytes contain almost diploid nuclei [71]. Ducks and geese, which can fly without rest for about 10 h at an altitude of 8–10 km, with an oxygen concentration threefold lower than at sea level and air temperature of −40 °C, have cardiomyocytes with a high ploidy (6–8 genomes) [104]. At the intraspecies level, the association between the early-stage hypoxia and the increased cardiomyocyte ploidy was found in humans with tetralogy of Fallot and other congenital heart defects that mix arterial and venous blood [72,77,148].

Regeneration may include the ability of certain types of multinucleated polyploid cells to enhance stem properties. In addition, in rapidly renewing tissues with high proliferative potential, the multinuclear and binuclear polyploid cells can give rise to lower ploidy cells with manifestations of stemness. For example, in drosophila ovaries and testes, stem cells appeared from the amitotic division of polyploid cells containing 4–16 genomes [156]. The incentive for depoliploidization is stress, associated with starvation or aging [156]. In a sponge, totipotent stem cells are formed from binuclear polyploid precursors of tezocytes [157]. These data indicate that the relationship between polyploidy and stemness is evolutionary conserved. Notably, in evolution, the appearance of lower-ploidy cells from the amitotic division of a polyploid cell is considered as one of the mechanisms of the origin of multicellular organisms, called ‘cellularisation’ [158].

However, polyploidy-associated regeneration has also species-specific effects. For example, tetraploid cells of large mammals typically experience replicative aging after endoreplication errors, although tetraploid mesenchymal stem cells and heart interstitial cells of murine rodents (mouse, rat) avoid replicative aging [116]. Murine rodents have weaker cell cycle control compared to larger mammals due to the evolutionary balance in rodents being biased in favor of the rapid development at the expense of accuracy and reliability of cellular processes [159,160]. Therefore, they have a higher ability of regeneration due to the proliferation of tetraploid cells, however, this ability increases the likelihood of carcinogenesis.

Polyploidy can be an important regeneration mechanism during aging when the proliferative potential of diploid cells is insufficient to repair defects in DNA, cytoskeleton, mitochondria, and other cellular components resulting from the accumulation of molecular errors, oxidative stress, functional overloads, inflammation, and mechanical tissue damage. The relationship between polyploidization and aging was observed in the retinal epithelium, vascular epithelium, megakaryocytes, lymphocytes, neurons, and other cells [161]. In some cases, damage-induced polyploidization is only temporary salvation in an emergency. For example, in the epicardium of *Danio rerio*, after the completion of regeneration by polyploidization, the polyploid cells were purified by apoptosis and replaced by dividing epicardial cells [1].

In general, regeneration through polyploidization is most likely a necessary measure, which provides a safety margin when normal regeneration due to proliferation of diploid cells is not possible. It may be fraught with genomic instability leading to oncogenesis.

## 8. Conclusions

Polyploidy is widespread in whole organisms and individual cells of multicellular tissues. Organismal polyploidy is widely recognized as an important driver of evolution. By increasing the amount of information and the scope of genetic variation, it increases tolerance to a wide range of stressful conditions, promotes biological plasticity and adaptation to new ecological niches, and masks harmful effects of mutations. Somatic polyploidy exists in many tissues of multicellular organisms. In mammals, polyploid cells appear during normal postnatal organogenesis. The degree of polyploidization is mostly determined by functional load and pathologic stress during development.

The accumulation of genomes can also be a manifestation of oncogenesis, aging, inflammation, and other pathologic conditions. Extensive cross-species studies indicated that polyploidy, on the one hand, is associated with the decrease in organ functional potential and, on the other hand, it enhances plasticity and adaptation to survival under extreme conditions (particularly hypoxia). In other words, it mitigates a trade-off between adaptation and function. Therefore, polyploidy is an important factor of developmental programming of many adult diseases (including heart diseases, hypertension, neurodegenerative disease, type 2 diabetes, metabolic syndrome and others). Under genotoxic conditions, when entering in mitosis inevitably leads to cell death, polyploidy is an adaptive replacement for a completed cell cycle. Depending on mitotic potential of cells and tissues, polyploidy can be dangerous or beneficial. On the one hand, genome duplication is an evolutionarily conserved way of tissue regeneration and renewal; on the other hand, polyploidy is the first step towards the emergence of tumor cells and cells resistant to cytostatics. Notably, genes arising via evolutionary polyploidization (ohnologs) are heavily involved in cancer and other diseases.

Comparative transcriptomic studies indicated that organismal polyploidy in evolution and somatic polyploidy in development has common features, which are based on the adaptive changes in gene expression. They are the enhancement of evolutionarily ancient programs of unicellularity, morphogenesis, life extension, and the reorganization of energy metabolism. The bivalent genes allowing rapid switches between different cellular programs can be the key regulators, determining common features of organismal and somatic polyploidy (they are strongly enriched in both cases). We hope that the evidence provided in this review elucidate the role of polyploidy in evolution, development, aging, and carcinogenesis, and may contribute to new strategies for control of organogenesis that could promote regeneration and prevent cell transformation.

## Data Availability

All data are provided in the manuscript.
